# The extracellular phage-host interactions involved in the bacteriophage LL-H infection of *Lactobacillus delbrueckii* ssp. *lactis* ATCC 15808

**DOI:** 10.3389/fmicb.2013.00408

**Published:** 2013-12-24

**Authors:** Patricia Munsch-Alatossava, Tapani Alatossava

**Affiliations:** Department of Food and Environmental Sciences, University of HelsinkiHelsinki, Finland

**Keywords:** bacteriophage LL-H, lactic acid bacteria, *Lactobacillus delbrueckii*, lipoteichoic acid, phage receptor, antireceptor, phage adsorption, phage–host interaction

## Abstract

The complete genome sequence of *Lactobacillus* bacteriophage LL-H was determined in 1996. Accordingly, LL-H has been used as a model phage for the infection of dairy *Lactobacillus*, specifically for thermophilic *Lactobacillus delbrueckii* ssp. *lactis* host strains, such as ATCC 15808. One of the major goals of phage LL-H research consisted of the characterization of the first phage-host interactions at the level of phage adsorption and phage DNA injection steps to determine effective and practical methods to minimize the risks associated with the appearance and attack of phages in the manufacture of yogurt, and Swiss or Italian hard type cheeses, which typically use thermophilic lactic acid bacteria starter cultures containing *L. delbrueckii* strains among others. This mini review article summarizes the present data concerning (i) the special features, particle structure, and components of phage LL-H and (ii) the structure and properties of lipoteichoic acids (LTAs), which are the phage LL-H receptor components of *L. delbrueckii* ssp. *lactis* host strains. Moreover, a model of the first, extracellular, phage-host interactions for the infection of *L. delbrueckii* ssp. *lactis* ATCC 15808 by phage LL-H is presented and further discussed.

## INTRODUCTION

The food industry benefits from the use of microbes as “work horses” in food processing, which contribute to the texture, chemical, and sensory properties of final food products. Lactic acid bacteria (LAB) are perhaps the most common bacteria applied as starter cultures for food manufacture. At an industrial scale, LAB starter-based lactic fermentations are performed in tanks with volumes of up to tens of cubic meters. The high number of starter cells comprising a single or a few strains makes these types of food processes highly susceptible to sudden appearance and attack of bacterial viruses, that is (bacterio)phages, and consequently increases the risk of the failure to control food fermentations and the quality of food products. LAB phages and phage resistance in LAB starter cultures have been intensively studied for decades because of the economic impact of the phage problems on the food industry. To obtain an in-depth scientific basis for the development of tools and approaches to minimize the risks of LAB phage infections in industrial food preparations associated with LAB fermentations, a better understanding of the origin, genetic diversity, and evolution of phages and phage biology, including phage–host interactions and phage resistance mechanisms (Samson et al., 2013), is needed.

The bacteriophage LL-H was isolated in 1972 from a whey sample originating from a problematic Emmental cheese production lot at a co-operative cheese processing plant in Hauho (Finland). The cheese starter culture employed at this dairy contained the *Lactobacillus delbrueckii* ssp. *lactis* (formerly *L. lactis*) strain LL23, which is sensitive to phage LL-H infection (Alatossava and Pyhtilä, 1980). Presently phage LL-H is one of the most thoroughly studied LAB phages and the first *Lactobacillus* phage, for which the complete genome sequence has been determined (Mikkonen, 1996; Mikkonen et al., 1996). Subsequently in the research, strain LL23 has been replaced with ATCC 15808, a more phage LL-H sensitive and widely available strain compared to LL23 strain.

## STRUCTURE OF PHAGE LL-H PARTICLE

**FIGURE 1:**
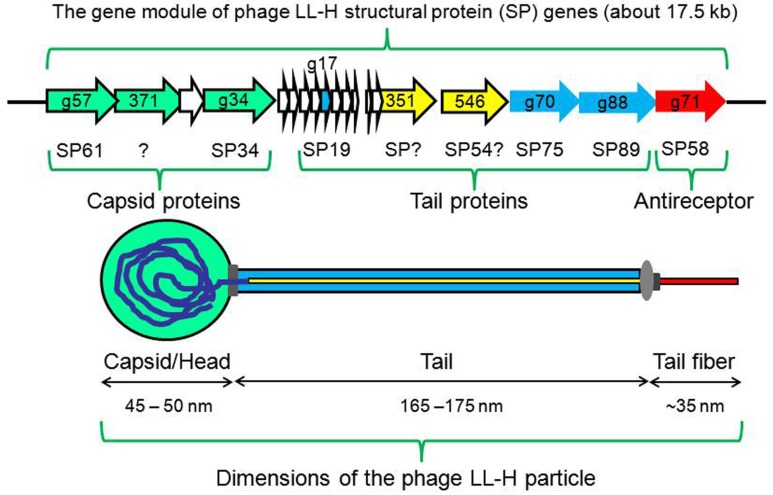
**Schematic diagram of phage LL-H particle.** Genes encoding capsid and tail proteins are indicated as green and blue, respectively. Gene *g71* (red) encodes the antireceptor protein SP58. The proteins coded by genes *orf351* and *orf546* (yellow) are considered as tape measure protein (TMP) homologs, likely localized to the core of the tail. The base plate is indicated as gray.

The results from electron microscopy (EM) studies on phage LL-H have revealed that this phage represents the most common morphological group among phages having an icosahedral head (capsid coat containing compactly packed linear phage DNA) and a long, non-contractile tail. A small base plate and a flexible tail fiber are located at the end of the tail, as summarized in **Figure 1** (Alatossava and Pyhtilä, 1980; Alatossava, 1987; Forsman and Alatossava, 1991; Forsman, 1994). Phage LL-H belongs to the *pac-*type phages comprising a linear ds-DNA molecule of approximately 38 kb with a 3 kb terminal repeat, packaged inside each phage capsid (Forsman and Alatossava, 1991). The linear LL-H DNA inside the capsid likely complexes with the divalent cations Ca^2^^+^ and/or Mg^2^^+^, which are co-transported into the cell as counterions of LL-H DNA during phage DNA injection (Alatossava, 1987; Alatossava et al., 1987). Phage LL-H particles are sensitive to Tris-buffer treatment (dialysis or gel filtration with Tris-buffer), which promotes *in vitro* phage DNA ejection (Alatossava, 1982). LL-H phages readily form clusters comprising filled and empty phage (ghost) particles connected together at the ends of the tails (Alatossava and Pyhtilä, 1980).

Based on the SDS-PAGE analysis of twice-purified (by CsCl density gradient centrifugation) LL-H phage particles, nine different structural proteins of 89, 75, 61, 58, 54, 34, 23, 19, and 14 kDa have been identified (Alatossava, 1987; Forsman and Alatossava, 1991). Among these, six phage structural proteins have been further characterized at the gene level (**Figure 1**): gene *g34* encodes the major capsid protein SP34; gene *g57* encodes the minor capsid protein SP61; gene *g17* encodes the major tail protein SP19; genes *g70* and *g88* encode the two minor tail proteins, SP75 and SP89, respectively; and gene *g71* encodes the tail fiber antireceptor protein SP58 (Gp71) (Trautwetter et al., 1986; Vasala et al., 1993; Mikkonen and Alatossava, 1994; Vasala, 1998; Ravin et al., 2002). No phage genes have been identified for the 54, 23, and 14 kDa LL-H structural proteins. These proteins may represent the proteolytic products of some other phage structural proteins or may be of bacterial origin. We re-sequenced the region between *orf351* and *orf360* described previously (Mikkonen and Alatossava, 1994; Mikkonen, 1996), and observed an additional G (GGG instead of GG) after the nt position 13888 (Mikkonen, 1996); consequently a single larger gene, *orf546,* was identified (**Figure 1**). Gene *orf546* could potentially encode the observed structural protein SP54 that is present in the phage LL-H particle but is absent in the LL-H ghost particle (Trautwetter et al., 1986). The N-terminal domain of phage LL-H ORF351 (aa-residues 20–105) shows amino acid sequence homology with the domains of several phage tape measure proteins (TMP) including *L. delbrueckii* phage c5 putative TMP (Riipinen, 2011; Riipinen et al., 2011). In addition, the C-terminal end of phage LL-H ORF546 containing peptidoglycan (PG)-hydrolysing domain features, shows aa sequence homology with a putative TMP of *L. delbrueckii* phage JCL1032 (Riipinen, 2011; Riipinen et al., 2011). Accordingly, both ORF351 and ORF546 proteins have been localized to the core of the phage LL-H tail, similar to TMPs (Plisson et al., 2007). However, instead of a single multifunctional, large TMP (Boulanger et al., 2008), the TMP-associated PG-hydrolysing and cytoplasmic membrane (CM)-binding activities preceding phage DNA transfer are mediated through separate TMP homologs, proteins ORF546 (SP54?) and ORF351, respectively, in phage LL-H (**Figure 1**).

## STRUCTURE AND PHAGE RECEPTOR PROPERTIES OF *Lactobacillus delbrueckii* LIPOTEICHOIC ACIDS

The first step of the phage infection cycle is the phage adsorption, the specificity of which is determined by the host bacterial surface component(s) and the phage component(s), receptor(s), and antireceptor(s), respectively. Furthermore, phage adsorption can be divided in two phases: a reversible phase and an irreversible phase. Among Gram-positive eubacteria, peptidoglycans, wall teichoic acids (WTA), lipoteichoic acids (LTA), and CM-associated proteins have been reported as phage receptor molecules. Proper genetic changes like spontaneous mutations affecting the structures of these molecules can prevent the adsorption of a particular phage and consequently increase the phage resistance of the mutated bacterial strain. However, the coevolution of phages with host bacteria facilitates mutations. For example, mutations in the antireceptor gene could change the host ranges of these mutant phages, and could potentially make the receptor mutant strains sensitive to the infections with these types of antireceptor phage mutants.

Both the phage receptor mutants of strain ATCC 15808 (the strain Ads-5) and the antireceptor mutants of phage LL-H (the strain LL-H-a21) have been isolated and further characterized (Ravin et al., 2002; Räisänen et al., 2004, 2007). The phage receptor mutant strain Ads-5 does not adsorb wild-type phage LL-H, but rather facilitates the effective adsorption of the host range phage mutants such as LL-H-a21. Each of the five host range phage mutants of LL-H studied contains a single nucleotide change at the 3′-end of gene *g71*, which encodes a mutant protein with a single aa substitution (Asn to Lys, Ala to Ser, or Gln to His) in the C-terminal end [in the region 380–543 aa of the 656 aa-protein SP58 (Gp71)]. Consequently, *g71* has been designated as the antireceptor-encoding gene. The structural analyses of LTAs from ATCC 15808 and Ads-5, the phage receptor mutant strain of ATCC 15808, have revealed a significant difference: the polyglycerolphosphate backbone of the LTA from Ads-5 lacks the single glucose moiety located most probably at the surface end of the LTA from ATCC 15808. Other structural features of LTAs, such as the levels of D-alanylation, average numbers of glycerolphosphate repeats, and the ratios and compositions of fatty acids linked to the triglucose moiety of the glycolipid anchor were not changed (Räisänen et al., 2007). The results of phage inactivation studies using purified LTA preparations suggest that the surface glucose substituted LTA is required for the specific reversible adsorption and enough free, nonsubstituted glycerol residues (allowing local negative charge in the LTA backbone) for the irreversible adsorption of wild-type LL-H. For the host range phage LL-H mutants, both the surface glucose-substituted and surface glucose-free forms of LTAs are equally functional as phage receptors (Räisänen, 2007). Thus, the extension of the host range may occur at the expense of the specificity of the phage receptor.

## A MODEL OF PHAGE LL-H ANTIRECEPTOR – HOST LTA INTERACTIONS

**FIGURE 2:**
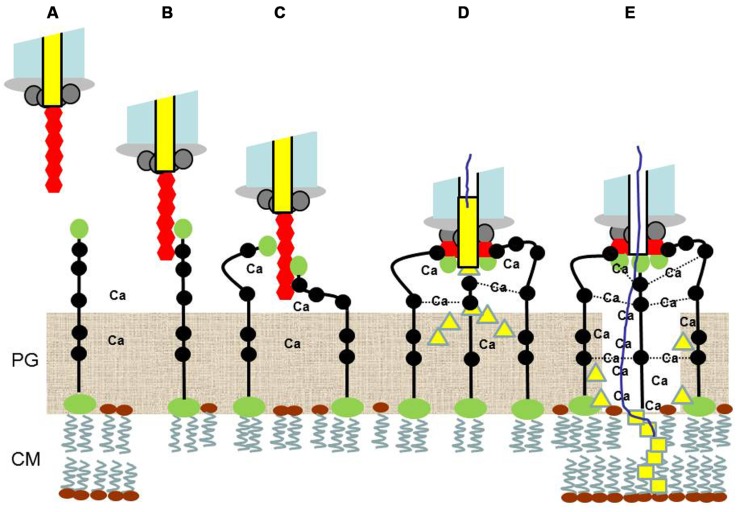
**A model of phage LL-H antireceptor-*Lactobacillus delbrueckii* ssp. *lactis* ATCC 15808 lipoteichoic acid (LTA) interactions during the extracellular phase of phage infection [steps (A–E)**]. **(A)** The LL-H tail fiber (red) is suggested to comprise six antireceptor protein (SP58) subunits. **(B,C)** Each antireceptor subunit initially interacts (reversible adsorption) with the top glucose moiety (green), followed by one of the negatively charged top phosphate groups (black) of LTA (irreversible adsorption) to facilitate the tail fiber attachment to the ends of up to six LTA molecules outside the peptidoglycan (PG) layer. **(D)** SP58 subunits-LTA interactions promote the tail fiber rearrangement into a ring structure attached to the end of the tail base for the release of the TMP homologs ORF546 (SP54?) and ORF351 from the tail core into the space restricted by the SP58-bound LTA molecules and further stabilized with calcium (Ca) bridges. **(E)** The PG-degrading activity of ORF546 proteins (yellow triangle) produces additional space for the formation of the stable Ca-LTA channel between the end of the tail base and the cytoplasmic membrane (CM). The Ca-LTA channel provides a gateway for ORF351 proteins (yellow square) to interact with the CM, and to further guide the transfer of the linear phage LL-H DNA complexed with calcium, through the CM into the cytoplasm of the infected host bacterial cell.

The structural properties of the phage LL-H particle and the genetic, biochemical, and electron microscopic data on the phage LL-H antireceptor/fiber, the ATCC 15808 LTA as phage receptor, and the phage LL-H infectivity and stability properties suggest, for the extracellular interactions between phage LL-H antireceptor and LTAs of the ATCC1 5808 host strain, a model described in **Figure 2**. The tail fiber, which is approximately 35 nm in length (Forsman and Alatossava, 1991; Forsman, 1994), is considered as a flexible hexapolymer of the antireceptor protein SP58 encoded by gene *g71* of phage LL-H. The C-terminal end of each antireceptor protein subunit contains one domain responsible for the reversible, specificity-determining binding to the surface end of the LTA, primarily mediated through hydrogen bonds to the glucose moiety. The second domain of the antireceptor protein SP58 ensures the irreversible binding to the negatively charged glycerol phosphate group(s) (no or low local D-Alanylation of glycerol phosphate repeats) close to the surface end of the LTA, possibly mediated through ionic bonds. Altogether, up to six LTA molecules can bind to the single phage tail fiber, promoting the rearrangement of the fiber to form a ring structure with a maximum diameter of 11 nm (i.e., 35 nm/π) attached to the tail base by the interactions of the six antireceptor protein subunits. The occurrence of the phage tail end-LTAs complexes could explain the observed heterogeneous structures and clusters of phage tail ends of negatively stained LL-H phage and empty LL-H ghost particles (e.g., Alatossava and Pyhtilä, 1980). The ring arrangement of the six antireceptor subunits suggests that the TMP homologs ORF546 (SP54?) and ORF351 are released from the core of the tail before the release of linear phage DNA. The muranolytic activity of ORF546 degrades PG inside the space of the LTA molecules (up to six) attached to the hexameric antireceptor (SP58) ring, facilitating the free movement of these attached LTA molecules and the subsequent formation of a stable calcium-LTA channel between the tail base end and CM as a tail extension. Phage LL-H infectivity is highly dependent on the external calcium or magnesium concentration, with an optimum of nearly the Ca/Mg solubility limits of the medium, 20–40 mM, which is approximately one log-unit higher than the Ca/Mg optimum required for phage LL-H adsorption (Alatossava, 1987). Accordingly, the requirements for Ca-LTA channel formation could reflect the observed high external calcium (or alternatively magnesium) concentration for optimal phage LL-H infectivity. Moreover, the proposed Ca-LTA channel would act as a gateway to the CM for ORF351, which contains two putative transmembrane motifs (residue regions 140–170 and 250–270) following the N-terminal tape measure domain. These interactions are likely required for the successful and effective transfer of linear phage DNA as a Ca-complex through the CM into the cytoplasm. The observed m.o.i. (multiplicity of infection)-dependent influx of calcium into the infected cell during the first minutes of phage infection (Alatossava et al., 1987) supports this model.

## PERSPECTIVE

A model of the extracellular interactions between the phage antireceptor and LTA receptors of the host has been proposed. The suggested formation of a stable calcium-LTA channel as the connecting structure between the phage LL-H tail base and the host CM could reflect the observed high calcium dependency for optimal LL-H infectivity. The Ca-LTA channel formation could exist not only in the case of phage LL-H, but also more generally among Gram-positive Ca-dependent phages, which do not contain phage lysin as an external structural tail base component.

## Conflict of Interest Statement

The authors declare that the research was conducted in the absence of any commercial or financial relationships that could be construed as a potential conflict of interest.
